# Retraction: Investigating the relationship between insulin use and all-cause mortality, breast cancer mortality, and recurrence risk in diabetic patients with breast cancer: A comprehensive systematic review and meta-analysis

**DOI:** 10.1371/journal.pone.0339149

**Published:** 2025-12-18

**Authors:** The PLOS One Editors

The *PLOS One* Editors retract this article [1] due to concerns about potential manipulation of the publication process. These concerns call into question the validity and provenance of the reported results and compliance with PLOS policies. We regret that the issues were not identified prior to the article’s publication.

MVL, MM, SK, and AMH did not agree with the retraction. RC either did not respond directly or could not be reached.
